# Developing an integrated rehabilitation model for thoracic cancer services: views of patients, informal carers and clinicians

**DOI:** 10.1186/s40814-018-0350-0

**Published:** 2018-10-18

**Authors:** Joanne Bayly, Bethany M Edwards, Nicola Peat, Geoffrey Warwick, Ivo M Hennig, Arvind Arora, Andrew Wilcock, Irene J Higginson, Matthew Maddocks

**Affiliations:** 1 Cicely Saunders Institute of Palliative Care, Policy and Rehabilitation, Kings College London, Bessemer Road, Denmark Hill, London, SE5 9PJ UK; 2 Guy’s & St Thomas’s NHS Foundation Trust, London, UK; 3 King’s College Hospital NHS Foundation Trust, London, UK; 4 Nottingham University Hospitals NHS Trust, Nottingham, UK; 5 University of Nottingham, Nottingham, UK

**Keywords:** Lung cancer, Mesothelioma, Focus groups, Rehabilitation, Qualitative, Intervention development, Feasibility trial

## Abstract

**Background:**

Access to rehabilitation to prevent disability and optimise function is recommended for patients with cancer, including following cancer diagnosis. Models to integrate rehabilitation within oncology services as cancer treatment commences are required, but must be informed by those they are intended to support. We aimed to identify views of patients, carers and clinicians to develop and refine a rehabilitation model to be tested in a feasibility trial for people newly diagnosed with lung cancer or mesothelioma.

**Methods:**

We conducted a focus group study with people affected by lung cancer or mesothelioma, their carers and clinicians providing their care to identify priorities for rehabilitation in this period. We sought views on core intervention components, processes and outcomes and integration with oncology services. Data were analysed using thematic analysis.

**Results:**

Fifteen clinicians (oncologists, nurse specialists, physiotherapists and occupational therapists), nine patients and five carers participated. A proposed outline rehabilitation model was perceived as highly relevant for this population. Participants recommended prompt and brief rehabilitation input, delivered whilst people attend for hospital appointments or at home to maximise accessibility and acceptability. Participants recognised variation in need and all prioritised tailored support for symptom self-management, daily activities and the involvement of carers. Clinicians also prioritised achieving fitness for oncology treatment. Patients and carers prioritised a sensitive manner of approach, positivity and giving hope for the future. Participant’s recommendations for outcome measurement related to confidence in usual daily activities, symptom control and oncology treatment completion rates over objective measures of cardiorespiratory fitness.

**Conclusion:**

The importance of providing tailored rehabilitation around the time of diagnosis for people with lung cancer or mesothelioma was affirmed by all participants. The refined model of rehabilitation recommended for testing in a feasibility trial is flexible, tailored and short-term. It aims to support people to self-manage symptoms, tolerate cancer treatments and to remain active and independent in daily life. It is delivered alongside scheduled hospital appointments or at home by an expert practitioner sensitive to the psycho-social sequelae that follow a diagnosis of thoracic cancer.

**Electronic supplementary material:**

The online version of this article (10.1186/s40814-018-0350-0) contains supplementary material, which is available to authorized users.

## Background

**Fig. 1:**
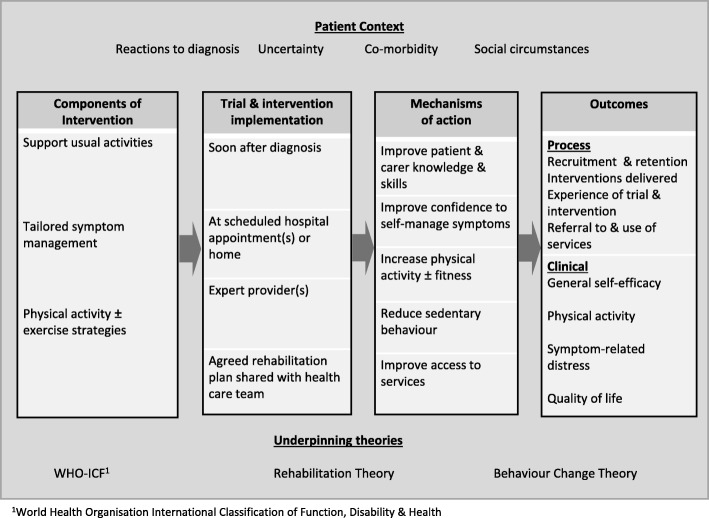
Outline rehabilitation logic model [14]

Rehabilitation following a diagnosis of cancer is recommended to help people retain their functional independence during and after oncology treatment and to mitigate subsequent disability [1]. Rehabilitation is defined as "a set of measures that assist individuals who experience, or are likely to experience, disability to achieve and maintain optimal functioning in interaction with their environment". (http://www.who.int/disabilities/brochure_EN_2.pdf?ua=1). National and international cancer guidelines recommend that it is offered from the point of diagnosis. Nonetheless, rehabilitation services are not always integrated into cancer services, and there is a lack of data on the feasibility and acceptability of rehabilitation models, particularly in advanced cancer [2]. New models are needed as despite compelling evidence of need relating to the physical, psychological, social and functional consequences of diagnosis and treatment [3–6], people with thoracic cancer may not be willing to access services [7–10]. Rehabilitation trials in this population are predominantly exercise or symptom self-management based and have not directly addressed participation in daily life activities in the period following diagnosis when people are at risk of deconditioning [11]. To begin to address this, based on the literature, we developed outline parameters for a comprehensive model of rehabilitation. These included integration with oncology services, delivery soon after diagnosis and tailored components to support people maintain participation in daily activities and minimise the onset of impairments as they commence cancer treatment (see Fig. 1). To support identification of environmental and personal factors which may act as barriers to participation in rehabilitation [9, 12, 13], determinants or mechanisms of effect [14], the outline model was underpinned by the World Health Organisation International Classification of Function Disability and Health, [15] plus theories of rehabilitation [16] and behaviour change (see Fig. 1) [17–19].

This outline model of rehabilitation meets the definition of complexity as defined in the Medical Research Council framework for the development and evaluation of complex interventions [20]. Thus, as part of the intervention modelling and development, we conducted this qualitative study to address key uncertainties relating to the model prior to testing it in a future randomised feasibility trial [21]. Recruiting patients to randomised controlled trials (RCTs) is challenging, and prospective identification of issues relevant to their successful conduct is recommended [20]. We therefore aimed to elicit patient, family and clinician views to further develop and refine the outline rehabilitation model. We also aimed to elicit factors that may facilitate or hinder participation in a feasibility trial of a rehabilitation intervention, including priorities for rehabilitation components and processes, trial outcomes valued by patients and clinicians and potential mechanisms of action. We also sought preferences for the trial delivery, including how to align activities with usual oncology and/or palliative care services.

## Method

### Design

This qualitative study sits within the development phase of an overarching sequential exploratory mixed-methods project. It is reported in accordance with the consolidated criteria for reporting qualitative research (COREQ) guidance [22] [see Additional file 1]. Focus groups were conducted with an action-focused pragmatic design. This methodology provided an efficient, economical method for obtaining participants’ views on health services where individual phenomenological data were not required [21, 23]. The facilitated group discussions promoted interactions between participants, allowed researchers to interact and respond to participants’ comments and potentially revealed richer and clarified data compared to individual interviews or questionnaires [24, 25]. Being part of a group discussion may also have improved respondent’s willingness to share their views [26].

### Participants and setting

Purposive sampling was used to obtain views from across two target groups; the first included those living with thoracic cancer (any primary lung cancer or pleural mesothelioma), informal carers, and members of patient support organisations focusing on thoracic cancer, and the second included oncologists, chest physicians, lung cancer and palliative care clinical nurse specialists (CNS), allied health care professionals (AHPs), social workers and psychologists with an interest in thoracic cancer. We aimed to recruit 12–15 participants from each population group (30 in total). Given the focused nature of our study within this specified population, the communication skills of the focus group facilitators and the cross-sectional nature of our analysis plan, we considered that this sample size would provide sufficient “information power” [27] to meet the aims of the study within a pragmatic time frame. We expected to undertake three focus groups per population group due to the logistical constraints experienced by clinicians and patients undergoing investigations and treatments. We did not intentionally seek data saturation [28].

Participants were invited to participate via public adverts, distributed via patient support organisations, cancer information centres, clinical special interest groups and oncology and rehabilitation services in the sites (one site in the Midlands and two in the London region, UK) planned for the randomised feasibility trial. Participants had to be willing to travel to a study venue and join a group discussion in English (via interpreters if available) for approximately 90 min. Travel costs were reimbursed, but no payment was provided for participation. Patients treated with radical surgery were excluded as the initial intended focus of the intervention was on patients on a non-curative treatment pathway, who represent the majority of people with thoracic cancer.

### Data collection

A systematic review [11] and discussions within the research team and a patient and public involvement (PPI) forum informed the development of the topic guide (see Additional file 2). Prior to attendance, participants were sent and asked to consider four short written patient stories, based on the literature and the first author (JB’s) clinical experience as a palliative care physiotherapist. These featured patients with varied functional impairments and concerns, to highlight the range of need in people with lung cancer or mesothelioma. They were designed to inform and stimulate group discussion. Importantly, they provided a mechanism for participants to discuss hypothetical scenarios, as well as or instead of their own experience, [29, 30] to help us identify rehabilitation components and service processes needed to best support people manage symptoms and remain active and independent throughout cancer treatment.

During each focus group, two researchers (JB and MM, BE, SP or MD) facilitated discussions on key components and strategies that should be included in the rehabilitation service, how the service should be delivered, including integration with other services, issues the research team need to consider when inviting people to join the trial that might influence their decision to participate and domains for trial outcomes. JB and MM are physiotherapists with experience supporting people with advanced cancer accessing palliative care services. JB, MM, BE, SP and MD have experienced facilitating focus groups and interview studies in oncology and palliative care research. Field notes were taken to allow for cross-checking with audio-recordings, but were not used directly in the analysis. All participants were given an opportunity to ask the researchers questions relating to the participant information sheet on arrival at the focus group, prior to written informed consent. The researchers had no prior relationship with patient or informal carer participants. Recruitment took place between January and September 2017 at three centres, two in London and one in the Midlands. We did not conduct member checking as extracting individual participant data from the group data alters the context of the utterances and may alter the meaning [31].

### Data analysis

Group discussions were audio recorded, transcribed verbatim and analysed using thematic content analysis [32]. Initial themes with potential to refine components of the rehabilitation service, trial processes and outcomes were identified from a preliminary review of the transcripts (JB). Transcripts were then independently coded line-by-line by two researchers (JB, BE) and interpreted deductively to address the study aims with additional themes interpreted inductively [31]. NVivo 11 software (QSR International) was used to incorporate discussion content, different perspectives on a topic, and drawing together of themes. Summary data were analysed and refined following discussion (JB, BE, MM, IJH) to identify common and divergent issues. As we sought participants’ views to inform the core intervention components, implementation processes and valued outcomes for a future feasibility trial, sub themes were refined and final themes were organised under overarching categories described in the MRC Process Evaluation Guidance [14, 33]. We sought to develop our theories of change [34], to improve our understanding of contextual factors and potential mechanisms of effect that may interact with the intervention and processes during the feasibility trial. These factors are presented within each section of the results.

## Results

Fifty-two people (35 patients and informal carers, 17 clinicians) responded to the public advert. Two patient participants were members of patient support organisations. Reasons for non-participation included the following: 6 were unable to attend on scheduled date(s), 5 did not reply to contact following receipt of participation information leaflet, 5 were unable to travel or not feeling well enough to attend, 4 were not interested, and 3 were cured of disease and ineligible.

Twenty-nine participants consented and took part in eight focus groups ranging from 43 to 77 min, with a median of 60 min. Due to difficulties convening groups that all interested participants could attend, and to minimise the time that recruited patient participants had to wait to attend a group, four, rather than the planned three, focus groups were held with each population group. Fifteen clinicians (12 females/3 male, 2 oncologists, 4 physiotherapists, 3 occupational therapists, 3 lung cancer nurse specialists and 1 palliative care nurse specialist) with all the recruitment sites for the feasibility trial were represented. Nine patients and 5 informal carers (9 female/5 male) participated. Of the 9 patients, 6 had a diagnosis of lung cancer and 3 mesothelioma. One participant was about to commence first-line treatment, one had completed treatment and seven were receiving maintenance chemotherapy or immunotherapy.

**Fig. 2:**
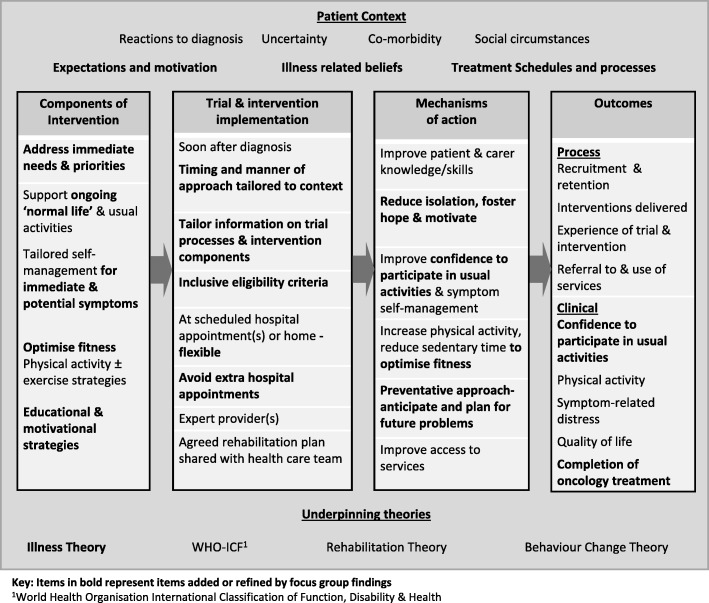
Refined rehabilitation logic model [14]

We present participant views that have informed and refined the design of the rehabilitation model [35, 36]. Nine themes are organised under three MRC Process Evaluation categories: (i) components of intervention, (ii) implementation of trial and intervention and (iii) outcomes. Factors relating to context and mechanisms of action are discussed within these three categories below and are presented separately in Figs. 1 and 2 that show the outline and refined rehabilitation model respectively.

### (i) Components of the rehabilitation intervention

Divergence was observed between patient/carer and clinician respondent views regarding the purpose of rehabilitation. Patients and carers emphasised that their main concerns following diagnosis were to get on with “normal life”, and they discussed how rehabilitation could help them “keep going”. Clinicians supported these aims but prioritised symptom management and physical activity as a means to support people to cope with oncology treatment, increase treatment options, and help patients complete a full treatment course and cope better with side effects. Despite this, responses from both groups confirmed that while the core components of the outline model (Fig. 1) should be retained (supporting usual activities; tailored symptom management and physical activity ± exercise), refinements were needed to maximise its acceptability to patients in the contextual period following diagnosis. It was recommended that although individual participants may need varying combinations of these components, an overarching educational and motivational approach were important components and potential mechanisms of action for everyone (see Fig. 2).

#### Supporting usual activities

Patients and carers recommended that intervention components help people address the impact of diagnosis and the effects of treatment on their normal life, rather than as a means to complete oncology treatment. They described the devastation and shock of diagnosis that can be a major threat to usual roles and activities. Components should help them feel safe and reduce concerns that they may do themselves harm. Even previously fit people may struggle to maintain normal routines and activities on hearing they have an illness they associate with death.

“…the other really important thing is to just keep your life... as normal as possible. I don’t know if a lot of people are managing that.” (PFG08 patient)

“…some people, I know of several, when they were diagnosed, they just gave up completely. One guy just sat in the chair, you know, wanting to die. His wife was distraught, because he’d been a very active person, so everybody reacts differently to the diagnosis.” (PFG04 patient)

This was also valued by clinicians who recognised such concerns exist and are not always addressed during clinic appointments, which focus on treatment planning.

“They don’t always tell us everything when they come into clinic. They’re just focused on discussing about chemotherapy, not everything. I feel that a lot of them, they don’t tell us. If we provide extra support and services I think they might just come out and tell, Oh, this is a problem I’m facing at home” 13, line. (CFG11 oncologist)

#### Tailored symptom management

In considering the aims for rehabilitation, participants reported that symptom management components must be tailored as the impact of symptoms on individuals varies considerably. Responses demonstrated that physical and psychological symptoms may precede diagnosis and impact on all aspects of daily life. Some reported feeling well initially, only developing symptoms during oncology treatment. Others were initially symptomatic, but symptoms resolved quickly following the start of treatment. The diversity highlighted the need for a detailed functional assessment and a personalised approach to support individuals to maintain participation in their normal roles and routines. Although functional limitation is highly prevalent at diagnosis in thoracic cancer, it should not be assumed.

“My fatigue started before my diagnosis. It started off with a dry cough, which just lasted for months and it didn’t go away. I started, you know, getting more and more fatigued. I couldn’t walk for more than a few yards without stopping for breath.” (PFG05 patient)

I couldn’t walk the kids to school, I couldn’t even walk to the end of the road really. Even walking upstairs was… you know and having breathing problems, you do panic sometimes. You can’t get your breath and it is trying to calm yourself down, especially if the kids are in the room and you don’t want to scare them. (PFG12 patient)

Management strategies should meet immediate needs when symptoms are present, but be offered alongside as a preventative approach. Patients, clinicians and carers agreed it was important to offer advice and training in anticipation of common experienced symptoms, as this may decelerate the impact of the disease, improve coping and reduce distress. This preventative approach was considered particularly important for people who either do not have symptoms, or where symptoms have responded to treatment but may reoccur.

Strategies discussed included advice and support for ongoing daily activity, general physical activity, exercise and dietary advice. Participants highlighted the physical symptoms of weakness, fatigue, breathlessness, weight loss, cough and pain, but also recommended that components address the psychological symptoms such as depression, anxiety and worries for the future.

“I think when you’re first diagnosed with cancer, all these symptoms that you’ve all talked about, if it’s not been explained to you that these are the feelings. But you see somebody like a physiotherapist or whatever, that can explain this to you and show you exercises that you can do at home to make you feel better, to make you feel you’re achieving something.” (PFG09 patient)

I’ve found that anxiety is quite high in a lot of those patients. Depression can be quite a prevalent symptom as well, but I think anxiety is the biggest, probably one of the biggest things you see at that stage. It impacts upon everything they do, simple things. They sometimes can’t do the simplest things because they’re worrying about what’s going to happen. (CFG08 AHP)

#### Optimising physical fitness

Participants recommended that rehabilitation should explicitly raise the importance of physical fitness and activity to help people cope with symptoms and treatment. Although this needs to be tailored, respondents considered it important to offer this to all patients, not just those with higher levels of fitness pre-diagnosis. Reflecting on their own experiences, patient participants thought that the language of fitness may foster hope at a time when much of the news being received is distressing, and may therefore improve the acceptability of the service.

Divergence on the role of fitness components was observed between clinicians and patient or informal carer participants. Clinicians emphasised the importance of optimising physical fitness for treatment and that improving it could expand options and increases completion rates.

“You’ve been given this diagnosis. We want to support you, so that you are well enough and as fit as you can be to go through whatever treatment they offer you, whatever that may be,” would be a good first step, because it would also maybe take their mind a little bit off the diagnosis and allow them to see the bigger picture. (CFG12 oncologist)

In contrast, patients and carers had not considered that participation may improve treatment outcomes. They recognised value in terms of promoting participation in everyday activities and tasks and carrying on with interests; indeed, those who had exercised were able to recall functional benefit. They recognised some people may find it hard to think about fitness at diagnosis, and small changes in behaviour, e.g. avoiding unnecessary rest, may be more achievable.

“It helps boost your whole system. You’re going to be less tired if you’re more active because the more you sit back and lay down, then you’re just going to get much weaker.” (PFG10)

#### A motivational approach

Patients and carers raised the importance of the attitude and behaviour of the health care professional delivering the service. As well as having insight and expertise to support people in distress, they need skills to encourage reluctant or poorly motivated people to participate. Family were also recognised as providing motivational support and participants recommended that trial participants are offered to invite a family member to be present during the intervention.

“You’ve got to tell me and you’ve got to keep prodding and getting me there. I might say, “I don’t want you, I don’t want you”, but I do. I realise I do.” (PFG06 patient)

“It is so easy just to sit there, go off to your treatments, come back home, sit there and [spouse] really encouraged me… “Why don’t you get up and do something, get everything working again”. I think I needed that push really, in the right direction.” (PFG12 patient)

Participants described that beliefs about cancer and its treatment cause some patients and their families to lose confidence to be active. They potentially limit themselves, even in situations where they may respond well to treatment and have months, if not years, to live. Participants recommended that professionals should recognise and sensitively address these beliefs to maximise engagement with the intervention components.

“One lady who, after her diagnosis, went home, wrote a will, gave up her job, started preparing for end of life, when actually two years down the line she’s still here with us.” (CFG03 CNS)

All participants recognised that some individuals need more encouragement than others to participate and that some would not be interested in rehabilitation.

“I think if you are in a palliative situation, it feels as if there’s more to take in, and there’s the, “Well, why bother?” kind of attitude, quite often, and overcoming that takes a bit of time.” (CFG12 oncologist)

Participants also described that multiple hospital and treatment appointments may influence how much time people felt they could give to participating in rehabilitation. They considered that self-management strategies are more likely to be used if they target meaningful priorities or goals and can be flexibly tailored to fluctuating health states.

“I have to be in the hospital all the time. I cannot go to work and I’m self-employed, do you understand? So all those things are changing. You lose everything in your head…You actually lose your confidence. (PFG11 patient)”

Promoting participants’ motivation and self-confidence was considered important, but strategies should be realistic in their scope. Some patients demonstrated resilience and were using self-directed strategies to manage symptoms and maintain fitness. In these situations, simple practical advice or affirmation of their own strategies may be all that is needed from a rehabilitation service.

…gentle exercise to keep you going and then maybe have another visit from physio or whatever and say, “Right, I have achieved this, I’ve done my 10,000 steps or whatever it is a day”. They say, “Right, that’s brilliant. So let’s up it then to 20,000 steps or try doing this...”. Encouraging them but pointing out... what you can do, what’s available for you to do to keep you fit. (PFG09 patient)

### (ii) Trial and intervention implementation

#### Trial screening and recruitment

When considering inviting people to join the feasibility trial, participants described that timing and manner of approach is important to consider. As described above, initial responses to diagnosis may be dominated by feelings of shock, distress and thoughts of death. It was agreed by all participants that usually, it would not be appropriate to introduce the trial on the day patients receive their diagnosis, when they may be overwhelmed trying to absorb large amounts of information and make decisions about treatment. Soon after this, appointment was preferred, with timing tailored to individual context. For those receiving their diagnosis as an outpatient, the subsequent appointment was preferred. Participants could see the advantages of waiting until treatment was planned. However, as the waiting period between diagnosis and treatment can be prolonged (several weeks), some patients may gain benefit from receiving rehabilitation before oncology treatment commences. Participants felt that screening contact and recruitment contacts could be made sooner if participants were diagnosed during an in-patient admission.

“…if it can improve your fitness during that period they might cope with the chemotherapy better. Patients are sitting at home thinking, ‘I haven’t started any treatment’. They’re worried. They always keep on thinking that they haven't started anything.” (CFG11 oncologist)

“I’m not absolutely sure that the first time that you attend for a consultant appointment, when you’ve got all other things on your mind, that that is necessarily the best time to be asked whether you want to join in all these things. You can’t put it into a context.” (PFG02 carer)

Linked closely to timing, the manner of approach was paramount for patients and their families. Some reported that initially they could only think about telling family members or getting things in order at home and preparing for death and may not have perceived the relevance of rehabilitation. They wanted those involved in screening and recruitment to present information about the service and the trial sensitively, demonstrating understanding about each person’s situation and discussing the trial in relation to how it might help them in their situation. Clinicians felt it was important to promote that the intervention as helping people get fit for treatment.

“Like [other participant] mentioned earlier, it’s about the manner. There should be a certain way to sit down with people and explain to them, because you’ve already given them really, really bad news. So if you could also explain to them in an educational way so that you don’t make them feel anymore worse than they already do. That will be quite helpful.” (PFG10 carer)

“I think after a decision has been made what treatment they want to go for, then having another session saying, Okay, you’ve decided to go for that treatment. Let’s get you as well as you can, so that you can go through that treatment and maintain your independence.” (CFG12 Oncologist)

Participants reported patients may feel overwhelmed with information at diagnosis that is hard to absorb, especially written information which may get put away, unread. They recommended that information about the trial be paced and tailored to each person’s readiness to engage. Some patients and carers reported that they did not understand the need for the rehabilitation model to be trialled in a controlled manner, and that they would not have wanted to be allocated to care as usual. Others recognised that a trial may ascertain if the rehabilitation offers additional benefit to oncology treatment. On discussion, they recommended that a clear explanation of the reasons for randomisation should be provided in all trial information.

“It’s going to work anyway. It’s obvious, if someone is lying in bed ill and then someone is getting up and doing exercise, they are going to benefit from it. I don’t think you need to trial it. (PFG06) I’m with you there…yes.” (PFG08)

Despite the challenges in making the initial approach to potential participants, clinicians, patients and carers considered that the patients should be offered participation in the trial in the period following diagnosis, rather than waiting for first oncology treatments to be completed. Clinicians highlighted the value of delivering early rehabilitation to optimise fitness and well-being. They suggested tailoring information about the trial to individuals’ current health status. For those without functional needs, the trial could be presented as testing an intervention that aims to get people fit for treatment. For those who are unwell, the trial could be presented as testing an intervention to help people manage the symptoms and limitations.

With the benefit of hindsight, patients described how the changes in their health status between diagnosis and treatment may have impacted on their engagement with a trial. Some, who felt well at diagnosis and did not anticipate how they would feel once treatment started, reported they may have declined to participate later on. Those whose health condition was deteriorating rapidly at diagnosis reported that whilst they may have declined to participate in a strenuous exercise intervention, they were open to an intervention to meet their immediate needs or feel more positive about their future.

“I think you would have struggled to listen to someone telling you to be active at that stage. You just couldn’t could you? (PFG013 carer) But the information I did get I think was really good from the guy. (PFG012 patient) Oh, the breathing and that, yes.” (PFG013 carer)

We had planned to include only patients with inoperable disease. However, clinical participants reported that patients having surgery would also benefit from this type of rehabilitation model. They recommended an inclusive eligibility criterion that includes patients on any oncology or palliative care treatment pathway.

“Then the people after surgery might struggle as well. I can’t understand why one group is in, it all and not the other, the one on chemo-radiotherapy. I think … involve everybody”. (CFG11) “Absolutely do, yes.” (CFG09 CNS)

#### Intervention delivery

The strongest message to come from all participants was for a flexible rehabilitation service, integrated with scheduled hospital and oncology treatment appointments. This requires the model to be tailored around participants’ schedules, their health state and preferences. Necessities, such as the need to continue working or look after family, require some patients to continue with usual roles and responsibilities. Clinicians reported an increase in “never smoked” working adults, some caring for children. Three of the patient participants were caring for school age children. A flexible approach to service delivery may improve accessibility for patients with family commitments.

“I kind of didn’t change my routine. I mean I’ve got three small children… You don’t know what to think, but I went into action. I thought I’m not going to let this conquer me. I’m stronger than this. I’m going to fight this.” (PFG08 patient)

To maximise inclusivity, the rehabilitation should be offered to participants in the location of their preference, which may be a hospital, at a schedule appointment, or in their own home. Participants and clinicians spoke of the boredom experienced by patients attending multiple appointments, waiting for hours in clinic or on treatment. Participants would prefer to receive the rehabilitation during these waiting times than come back for additional appointments. For others, travel itself was burdensome due to distance or overall health, and in these circumstances, home was the preferred location. Additionally, some patients and carers felt they would find it easier to learn rehabilitation techniques in their home environment. They considered that this flexibility would maximise participants’ willingness to join and remain on trial.

“I would say the vast majority of patients voice to us that they don’t want to come up any extra. I don’t think you will get them turning up unless it is on a day that they have already got an appointment. That is a big one.” (CGF06 AHP)

“…if someone was to approach you when you’re sitting there waiting for that oncologist. Is hours we’ve sat in waiting rooms. (PFG06 patient) It’s tiring as well, so if you’re kept busy then it’s not going to be that bad.” (PFG10 carer*)*

Clinicians felt that the proposed three contacts were appropriate and these were sufficient to address peoples’ functional needs following diagnosis without adding additional undue burden. Patients and carers were more concerned with not returning to the hospital for extra appointments than the number of contacts received during the intervention. However, they reported that some patients may want more prolonged involvement with rehabilitation services. Patient participants were happy with the proposed plan for referral to ongoing services, but were unsure about where or what these may be. Clinicians were also concerned about the availability of services for onward referral.

### Outcomes

#### Patient centred

All participants recognised the challenges of measuring the impact of the service where the natural course of the disease and overall health state in participants will be varied and unpredictable. Consequently, objective measures of fitness were not considered useful in such a broad target population.

“…it is so hard because of the population group. That is the thing because, you know, they just fluctuate so much, so getting any standardised assessment unless you are measuring something like their confidence in doing a task.” (CFG06 AHP)

Instead, participants recommended patient-reported outcome measures that assess changes in levels of symptom distress, physical activity, and function alongside confidence to perform daily activities and to manage symptoms. Clinicians also recommended using a quality of life measure.

“Perhaps they could have like a pre questionnaire, what does your daily routine involve today and then a follow up, what does your routine do today and compare them.” (PFG07 carer)

“rather than it being an actual symptom that may get better or get worse, it’s their confidence in managing their symptoms …a lot of it will be around them just feeling that they’ve got the skills, they’re equipped to manage on their own.” (CFG02 AHP)

#### Intervention process

All participants recommended measuring the nature and number of rehabilitation components, the frequency, mode and location of contacts, as well as experience and satisfaction with rehabilitation.

“I would think that, probably the group that felt it was not doing them any good would tend to start staying away.” (PFG05 patient)

#### Oncology treatment related

In addition to the number of participants joining and completing the trial, clinicians recommended assessing whether oncology treatments were received as planned, reasons for discontinuing treatment, treatment toxicity scores, unplanned hospital admissions and use of other health care services during the trial period.

They might have, I think, better tolerance for the treatment as well. I think they can tolerate better if they’re well supported psychologically and so on. Those kinds of things we include, the hospital stay, for example, what is their outcome and whether we had to reduce the dose of chemotherapy and so on, all those things we can do. (CFG11 oncologist)

“I don’t know if there are any independence scores that assess how independent patients stay, and compare that with the toxicity profile that we get from the chemotherapy, so you can see that patients react differently to the chemotherapy?” (CFG12 oncologist)

## Discussion

We conducted focus groups to develop and refine a complex intervention and procedures for a future randomised feasibility trial. The findings have illuminated factors to be refined in the conduct of a planned trial in this population. Implications include an improved understanding of contextual factors and mechanisms of action relating to intervention components, specific trial processes, and the selection of feasibility and clinical outcomes. The findings redirect the focus of the rehabilitation model towards helping people stay active and independent as they commence treatment by addressing beliefs, symptoms and psychological concerns that limit participation in daily life. Intervention components should include strategies to address needs in patients across the age range and with varying performance status. Recognising that people are often overwhelmed at diagnosis, information given should be individualised and brief, with strategies focused on immediate needs. The service should work towards meaningful goals, including improved capacity to tolerate oncology treatments. For people who feel well and have higher levels of fitness, a positive training approach should support people maintain independence and get the most from planned oncology treatments. This pro-active approach should seek to improve participants’ self-confidence to cope and address illness understandings, belief and fears that impact on function. It should support ongoing participation in usual activities and reduce sedentary time to maintain independence and fitness. This study confirms findings from previous research that patients place importance on how interventions are delivered [37]. Encouraging and supporting usual habitual activities wherever possible should align with patients’ desires to keep life normal, to not feel ill. Tailored exercise strategies can be added for those who are motivated. Strategies to manage common symptoms should be offered to all patients. This should provide immediate relief for those experiencing symptoms and include preventive and proactive strategies for those at risk of developing them during or following treatment. Previous rehabilitation studies in this population group have focused on discrete interventions, such as breathlessness management or exercise to address impairments [11]. However, beliefs pertaining to potential harm may preclude participation in rehabilitation, including exercise [9, 12, 13]. If symptoms are not present, people may see no value in participating in symptom self-management [10, 38].

Our findings corroborate findings of previous qualitative studies. The intervention and the trial processes should be flexible, integrating with usual oncology services to reduce the barriers that arise when patients are required to attend additional hospital appointments [9, 10]. Home-based appointments may improve recruitment and retention. Our trial eligibility criteria were refined because of the focus groups. The trial should recruit patients on any treatment pathway to maximise recruitment but also provide equitable care. The importance placed on the interpersonal attributes of the researchers has implications for all members of a feasibility trial research team. Those involved in screening should approach potential participants with sensitively, with an understanding of their unique context. Information about the trial can be paced during the screening and informed consent process, using a one-sided summary flyer, followed by the full participant information leaflet for those requesting further information.

Clinical outcomes should focus on domains meaningful to the varying needs of patients.

Process outcomes should include treatment completion rates, important to clinicians and patients. Although challenging, our findings suggest that delivering interventions following diagnosis, is warranted [39–41], and we, like others, [42] suggest that integrating into existing services and modelling services with close involvement of all involved will facilitate future practice change.

Our initial development work for the trial intervention was underpinned by models and theories of rehabilitation [16] and behaviour change (The Behaviour Change Wheel [17], Intervention Mapping [18] and Implementation Intention Planning [19]). However, our findings suggest that these theories alone may not have the best “ecological fit” [35] in the context of a new diagnosis of thoracic cancer. Importantly, these theories may not illuminate potential mechanisms of action relating to the sudden change in circumstances or uncertainties experienced by patients and their families. As a result, the intervention likely has a reduced scope of action.

Theories of illness provide explanations for contextual factors such as the tensions experienced by patients who want to remain well and continue with normal life yet struggle to prioritise rehabilitation. Reorientation of self-identity and adjustment takes effort and time, varying depending on the context of each person and the approach taken by the clinicians providing their care [43–45]. The manner of approach and the focus of rehabilitation contacts should be context sensitive and tailored, addressing all possible mechanisms of action, including those relating to experiences of illness. These will include the “immediacy” [44] of participants’ current life experience, their pressing needs, concerns and beliefs following diagnosis. Use of illness theory will support refinement of the rehabilitation model so these factors are addressed in the intervention design, implementation and evaluation.

### Strengths and limitations

The focus group study has refined the underpinning theory, design and implementation processes of a rehabilitation model for testing in a future feasibility trial [46]. Participants were recruited from all proposed trial sites and clinical groups who will be referring patients to the trial. We recruited patients (with diagnoses of both lung cancer and mesothelioma) and informal carers with a diversity of ages and life situation. The interaction between participants generated rich data concerning the variation in performance status, impairments and functional expectations present in this population. One limitation in our sampling is the possibility that only fitter patients interested in rehabilitation were recruited. They may not represent views of people who are more unwell. However, participants reported experiences of patients with lower levels of fitness and motivation and this has influenced our planned screening strategies and the intervention components. Challenges were experienced getting participants to focus groups on the same day resulting in two patient focus groups of three participants and one with two, which potentially limited participant interaction [28]. However, in these cases, participants engaged with each other and revealed unique perspectives not discussed in larger focus groups. Again, these contributed to the development of the themes during analysis. The action orientated pragmatic nature of this study meant we did not analyse latent themes [32] or consider wider structural issues which may impact on the implementation and outcomes of the intervention [47]. However, the findings have broadened our theoretical perspective and improved our rehabilitation model to improve the quality and evaluation of the planned randomised feasibility trial (ISRCTN92666109).

## Conclusion

The focus group findings provide insights for the development of rehabilitation interventions in patients with lung cancer and mesothelioma, which were used to refine the intervention content and delivery, working processes and target outcomes for a future feasibility trial. The rehabilitation intervention builds on the findings of earlier studies and is modelled using patient, carer and clinician views around delivery. It is inclusive, addresses a range of functional limitations and aims to integrate with oncology services. Beyond these direct implications, our findings can help inform the development of pro-active rehabilitation interventions in other populations newly diagnosed with advanced disease.

## Additional files

Additional file 1: Consolidated criteria for reporting qualitative studies (COREQ): 32-item checklist. (DOCX 17 kb)

Additional file 2: Focus group topic guide. (DOCX 17 kb)

## References

[1] Stout Nicole L., Silver Julie K., Raj Vishwa S., Rowland Julia, Gerber Lynn, Cheville Andrea, Ness Kirsten K., Radomski Mary, Nitkin Ralph, Stubblefield Michael D., Morris G. Stephen, Acevedo Ana, Brandon Zavera, Braveman Brent, Cunningham Schuyler, Gilchrist Laura, Jones Lee, Padgett Lynne, Wolf Timothy, Winters-Stone Kerri, Campbell Grace, Hendricks Jennifer, Perkin Karen, Chan Leighton (2016). Toward a National Initiative in Cancer Rehabilitation: Recommendations From a Subject Matter Expert Group. Archives of Physical Medicine and Rehabilitation.

[2] Granger CL (2016). Physiotherapy management of lung cancer. J Phys.

[3] Bayly JL, Lloyd-Williams M (2016). Identifying functional impairment and rehabilitation needs in patients newly diagnosed with inoperable lung cancer: a structured literature review. Support Care Cancer.

[4] Maguire R (2013). A systematic review of supportive care needs of people living with lung cancer. Eur J Oncol Nurs.

[5] Tishelman C (2010). Are the most distressing concerns of patients with inoperable lung cancer adequately assessed? A mixed-methods analysis. J Clin Oncol.

[6] Moore S, Darlison L, Tod AM (2010). Living with mesothelioma. A literature review. Eur J Cancer Care.

[7] Fitch MI, Steele R (2010). Supportive care needs of individuals with lung cancer. Can Oncol Nurs J.

[8] Brown Natasha M. K., Lui Chi-Wai, Robinson Peter C., Boyle Frances M. (2014). Supportive care needs and preferences of lung cancer patients: a semi-structured qualitative interview study. Supportive Care in Cancer.

[9] Cheville AL (2017). How receptive are patients with late stage cancer to rehabilitation services and what are the sources of their resistance?. Arch Phys Med Rehabil.

[10] Ellis J (2012). Considerations in developing and delivering a nonpharmacological intervention for symptom management in lung cancer: the views of patients and informal caregivers. J Pain Symptom Manag.

[11] Bayly J, et al. Changing health behaviour with rehabilitation in thoracic cancer: a systematic review and synthesis. Psychooncology. 2018;27(7):1675–94. 10.1002/pon.4684 29476566

[12] Granger CL (2017). Understanding factors influencing physical activity and exercise in lung cancer: a systematic review. Support Care Cancer.

[13] Granger Catherine L., Denehy Linda, Remedios Louisa, Retica Sarah, Phongpagdi Pimsiri, Hart Nicholas, Parry Selina M. (2016). Barriers to Translation of Physical Activity into the Lung Cancer Model of Care. A Qualitative Study of Clinicians’ Perspectives. Annals of the American Thoracic Society.

[14] Moore GF (2015). Process evaluation of complex interventions: Medical Research Council guidance. bmj.

[15] Organisation, W.H (2001). The international classification of functioning, disability and health: ICF.

[16] Wade D (2016). Rehabilitation – a new approach. Part three: the implications of the theories. Clin Rehabil.

[17] Michie S, Atkins L, West R (2014). The behaviour change wheel: a guide to designing interventions.

[18] Kok G (2016). A taxonomy of behaviour change methods: an intervention mapping approach. Health Psychol Rev.

[19] Hagger MS, Luszczynska A (2014). Implementation intention and action planning interventions in health contexts: state of the research and proposals for the way forward. Appl Psychol Health Well Being.

[20] Craig P (2008). Developing and evaluating complex interventions: the new Medical Research Council guidance. Bmj.

[21] O'Cathain A (2013). What can qualitative research do for randomised controlled trials? A systematic mapping review. BMJ Open.

[22] Tong A, Sainsbury P, Craig J (2007). Consolidated criteria for reporting qualitative research (COREQ): a 32-item checklist for interviews and focus groups. Int J Qual Health Care.

[23] Cornish F, Gillespie A (2009). A pragmatist approach to the problem of knowledge in health psychology. J Health Psychol.

[24] Kitzinger J (1995). Qualitative research. Introducing focus groups. BMJ.

[25] Qualitative Research Practice (2014). A guide for social science students and researchers.

[26] Hudson Peter (2003). Focus group interviews: a guide for palliative care researchers and clinicians. International Journal of Palliative Nursing.

[27] Malterud K, Siersma VD, Guassora AD (2016). Sample size in qualitative interview studies: guided by information power. Qual Health Res.

[28] Carlsen B, Glenton C (2011). What about N? A methodological study of sample-size reporting in focus group studies. BMC Med Res Methodol.

[29] Schoenberg NE, Ravdal H (2000). Using vignettes in awareness and attitudinal research. Int J Soc Res Methodol.

[30] Parry R, Land V, Seymour J (2014). How to communicate with patients about future illness progression and end of life: a systematic review. BMJ Support Palliat Care.

[31] Varpio L (2017). Shedding the cobra effect: problematising thematic emergence, triangulation, saturation and member checking. Med Educ.

[32] Braun V, Clarke V (2006). Using thematic analysis in psychology. Qual Res Psychol.

[33] Green J, Thorogood N. Qualitative methods for health research. London: Sage; 2013.

[34] De Silva MJ (2014). Theory of change: a theory-driven approach to enhance the Medical Research Council's framework for complex interventions. Trials.

[35] Moore GF, Evans RE (2017). What theory, for whom and in which context? Reflections on the application of theory in the development and evaluation of complex population health interventions. SSM Popul Health.

[36] Wells M (2012). Intervention description is not enough: evidence from an in-depth multiple case study on the untold role and impact of context in randomised controlled trials of seven complex interventions. Trials.

[37] Farquhar MC (2014). Is a specialist breathlessness service more effective and cost-effective for patients with advanced cancer and their carers than standard care? Findings of a mixed-method randomised controlled trial. BMC Med.

[38] Wagland R (2012). Considerations in developing and delivering a non-pharmacological intervention for symptom management in lung cancer: the views of health care professionals. Support Care Cancer.

[39] Cheville AL (2017). Cancer rehabilitation: an overview of current need, delivery models, and levels of care. Phys Med Rehabil Clin.

[40] Silver JK (2017). Integrating rehabilitation into the cancer care continuum. PM R.

[41] Robb K, Davis J (2015). Examining progress in cancer rehabilitation: are we closer to parity of esteem?. Eur J Cancer Care.

[42] Granger Catherine L., Parry Selina M., Denehy Linda, Remedios Louisa (2018). Evidence, education and multi-disciplinary integration are needed to embed exercise into lung cancer clinical care: A qualitative study involving physiotherapists. Physiotherapy Theory and Practice.

[43] Carel H. Phenomenology of illness. First ed. Oxford: Oxford University Press; 2016.

[44] Toombs SK. The meaning of illness: a phenomenological account of the different perspectives of physician and patient Volume 42. Dordrecht, Boston: Springer Science & Business Media; 1993.

[45] Charmaz K (1995). The body, identity, and self. Sociol Q.

[46] Levati S (2016). Optimisation of complex health interventions prior to a randomised controlled trial: a scoping review of strategies used. Pilot Feasibility Stud.

[47] Hawe Penelope (2015). Minimal, negligible and negligent interventions. Social Science & Medicine.

