# Supplementary material for: Developing an integrated rehabilitation model for thoracic cancer services: views of patients, informal carers and clinicians
Source: Pilot Feasibility Stud. 2018 Oct 18;4:160. doi: 10.1186/s40814-018-0350-0 (PMC6193311; doi:10.1186/s40814-018-0350-0)
Supplement: Supplementary file 2 — Focus group topic guide. (DOCX 17 kb) [file 40814_2018_350_MOESM2_ESM.docx]

**Additional file 2. Focus Group Topic Guide**

1. Thinking about the people in the stories & your own experiences
   can you tell us what day to day life is like for people in that time after they’ve been diagnosed?
   - 1. What might help people to manage, to stay active & independent?
     2. How would people feel being given information about practical things they can do now to help with their current symptoms and concerns?
     3. How would people feel discussing things they can do now to prevent symptoms, like weakness & breathlessness, developing in the future?

**The proposed rehab service:**1 to 3 appointments for patient & significant other with a physiotherapist or occupational therapist focusing on symptom management and physical activity to optimise function and participation in daily activities and roles.

1. Can you tell us your thoughts about how people might feel if offered this service?
2. How would it fit in with medical treatments and clinic appointments?
3. How might it help?
4. Does this kind of a service have a place in cancer care?
5. Can you foresee any problems, for instance what about after the service?
6. What do we need to think about when we are inviting people to join the research study?
7. What might influence whether people join the study?
8. We’ll ask people to complete questionnaires before and after the service. What kind of questions will help us find out if the service has been helpful or not?
   How long should the questionnaire be?
9. Is there anything you’d like to add about the new rehabilitation service or the research study that we haven’t covered?
